# Mutation spectrum of Kallmann syndrome: identification of five novel mutations across *ANOS1* and *FGFR1*

**DOI:** 10.1186/s12958-023-01074-w

**Published:** 2023-03-01

**Authors:** Guoming Chu, Pingping Li, Qian Zhao, Rong He, Yanyan Zhao

**Affiliations:** 1 Department of Clinical Genetics, Shengjing Hospital of China Medical University, Shenyang, 110004 Liaoning China; 2 Center of Reproductive Medicine, Department of Obstetrics and Gynecology, Shengjing Hospital of China Medical University, Shenyang, 110004 Liaoning China; 3 Department of Pediatric Urology, Shengjing Hospital of China Medical University, Shenyang, 110004 Liaoning China

**Keywords:** Kallmann syndrome, *ANOS1*, *FGFR1*, Splicing mutation, Minigene

## Abstract

**Background:**

Kallmann syndrome (KS) is a common type of idiopathic hypogonadotropic hypogonadism. To date, more than 30 genes including *ANOS1* and *FGFR1* have been identified in different genetic models of KS without affirmatory genotype–phenotype correlation, and novel mutations have been found.

**Methods:**

A total of 35 unrelated patients with clinical features of disorder of sex development were recruited. Custom-panel sequencing or whole-exome sequencing was performed to detect the pathogenic mutations. Sanger sequencing was performed to verify single-nucleotide variants. Copy number variation-sequencing (CNV-seq) was performed to determine CNVs. The pathogenicity of the identified variant was predicted in silico. mRNA transcript analysis and minigene reporter assay were performed to test the effect of the mutation on splicing.

**Results:**

*ANOS1* gene c.709 T > A and c.711 G > T were evaluated as pathogenic by several commonly used software, and c.1063-2 A > T was verified by transcriptional splicing assay. The c.1063-2 A > T mutation activated a cryptic splice acceptor site downstream of the original splice acceptor site and resulted in an aberrant splicing of the 24-basepair at the 5′ end of exon 8, yielding a new transcript with c.1063–1086 deletion. *FRFR1* gene c.1835delA was assessed as pathogenic according to the ACMG guideline. The CNV of del(8)(p12p11.22)chr8:g.36140000_38460000del was judged as pathogenic according to the ACMG & ClinGen technical standards.

**Conclusions:**

Herein, we identified three novel *ANOS1* mutations and two novel *FGFR1* variations in Chinese KS families. In silico prediction and functional experiment evaluated the pathogenesis of *ANOS1* mutations. *FRFR1* c.1835delA mutation and del(8)(p12p11.22)chr8:g.36140000_38460000del were assessed as pathogenic variations. Therefore, our study expands the spectrum of mutations associated with KS and provides diagnostic evidence for patients who carry the same mutation in the future.

**Supplementary Information:**

The online version contains supplementary material available at 10.1186/s12958-023-01074-w.

## Background

Idiopathic hypogonadotropic hypogonadism (IHH) is a rare genetic disease characterized by delayed or complete lack of puberty, which is caused by hypothalamic-pituitary–gonadal (HPG) axis dysfunction [1]. IHH is clinically divided into Kallmann syndrome with anosmia/hyposmia (KS) and normosmic IHH (nIHH). KS accounts for 50% of all IHH cases [2], and KS occurs in 1 per 30,000 men and 1 per 125,000 women [3].

KS is clinically heterogeneous, mainly manifesting as gonadal dysplasia and possibly accompanied by other congenital malformations, such as renal agenesis or hypoplasia, cleft lip/palate, dental agenesis, hearing impairment, bimanual synkinesis, or skeletal anomalies [1]. KS exhibits different inheritance patterns and genetic heterogeneity including X-linked recessive (*ANOS1*), autosomal recessive (*PROK2* and *PROKR2*), and autosomal dominant (*FGFR1*, *FGF8*, and *CHD7*) genes [4, 5]. *ANOS1*, located at the Xp22.3 and also known as Kallmann syndrome 1 (*KAL1*), is the first gene found to be associated with the X-linked form of KS [6]. Anosmin-1 protein that is encoded by *ANOS1* contains four domains: a N-terminal cysteine-rich region (CR domain), a whey acidic protein-like (WAP) domain, four fibronectin type III (FnIII) domains, and a histidine-rich C-terminal region [7]. Currently, more than 200 mutations in *ANOS1* have been included in the Human Gene Mutation Database (HGMD), all of which spread on the whole gene, and the number of mutation is increasing, but no mutation hotspots have been found in the affected regions [8].

Fibroblast growth factor receptor 1 (*FGFR1*) is located at 8p11.23. FGFR1 is a member of the tyrosine kinase superfamily of receptors. Dodé et al. reported that loss-of-function mutations of *FGFR1* underlay KS2 for the first time in 2003. KS2 is an autosomal dominant disease [9]. To date, more than 300 mutations in *FGFR1* have been included in HGMD, all of which included missense mutation, nonsense mutation, splice mutation, and rarely deletions. FGFR1 signaling plays an important role in neuronal migration, differentiation, survival, as well as cell proliferation during embryonic development mainly via the PI3K/Akt and MAPK pathways [10].

In this study, we recruited 35 unrelated individuals with IHH manifestations and identified three novel mutations in *ANOS1* and two novel mutations in *FGFR1*. Additionally, we verified the novel splice site mutation for transcription function; the c.1063-2 A > T mutation generated an abnormal transcript and translated into a truncated protein product. Thus far, more than 30 genes linked with IHH pathogenesis have been identified; however, the mutation spectrum is incomplete owing to the high genetic and clinical heterogeneity of KS [11]. Thus, our study expanded the mutation spectrum of KS and provided diagnostic evidence for patients with the same mutation in the future.

## Methods

### Participants

In total, 35 unrelated patients were recruited from Shengjing Hospital of China Medical University, all patients came for consultation because of disorder of sex development (DSD) (Table S1). In family 1, the proband (patient 1) manifested as small penis, cryptorchidism, and testicular dysplasia. In family 2, the proband (patient 2) phenotyped as small penis and testicular dysplasia. In family 3, the proband (patient 3) and his nephew showed cryptorchidism, left renal agenesis, and olfactory disorder. In family 4, the proband (patient 4) manifested as small penis, olfactory disorder, testicular dysplasia and polydactylism. In family 5, the proband (patient 5) phenotyped as cryptorchidism and testicular dysplasia.

### Genetic testing

Genomic DNA was extracted from peripheral blood samples using a QIAamp DNA Blood Mini Kit (QIAGEN, Germany) according to the manufacturer’s instructions. Custom-panel sequencing (575 genes covering endocrine-related diseases) and whole-exome sequencing (WES) were performed by MyGenostics Inc. (Beijing, China) (Table S1). After sequencing, the raw data were saved as a FASTQ format. Both Illumina sequencing adapters and low quality reads (< 80 bp) were filtered by cutadaptor software (http://code.google.com/p/cutadapt/). The clean reads were mapped to the UCSC hg19 human reference genome using the parameter BWA of Sentieon software.(https://www.sentieon.com/). The duplicated reads were removed using the parameter driver of Sentieon software, and the parameter driver is used to correct the base, so that the quality value of the base in the reads of the final output BAM file can be closer to the real probability of mismatch with the reference genome, and the mapped reads were used for the detection of variation. The variants of SNP and InDel were detected by the parameter driver of Sentieon software. Then, the data would be transformed to VCF format. Variants were further annotated by ANNOVAR software (http://annovar.openbioinformatics.org/en/latest/). In the present study, three steps were used to select the potential pathogenic variants in downstream analysis: (1) variant reads should be more than 5, and variant ration should be no less than 30%; (2) the variants should be removed, when the highest minor allele frequency (MAF) in 1000 Genomes, EXAC, ESP6500, gnomAD, and dbSNP was more than 5%; (3) the synonymous mutations should be removed, when they were not in the HGMD database. After that, the rest mutations should be the potential pathogenic mutations for further analysis.

The candidate causative gene mutations were verified through the Sanger sequencing. Target gene fragments including mutations were amplified using the PrimeSTAR® HS DNA Polymerase (#R010Q, Takara, Dalian, China). Sanger sequencing was performed on 3730 DNA analyzer (Applied Biosystems, USA) using BigDye™ Terminator v3.1 Cycle Sequencing Kit (Applied Biosystems, USA). Copy number variation-sequencing (CNV-seq) analysis was conducted using library construction kit (KR2000, Berry Genomics, Beijing, China) on the Illumina Nextseq CN500 platform. Sequencing data were analyzed by the data analysis system (Berry Genomics, Beijing, China).

### *ANOS1* mRNA transcript analysis

Total RNA was extracted from peripheral blood samples using a TransZol Up Plus RNA Kit (#ER501-01, TransGen Biotech Co., LTD, Dalian, Beijing, China) according to the manufacturer’s instructions. cDNA was synthesized from total RNA using PrimeScript™ RT reagent Kit with gDNA Eraser (#RR047A, Takara, Dalian, China). The cDNA products (NM_000216.4) were used as templates for PCR amplification using the following primers:

- F: 5′—CGAGTGGCTGCTGTGAATGTG—3′
- R: 5′—GCGAGTGGGTCGTCGTCTT—3′

*ANOS1* mRNA transcripts were identified by Sanger sequencing.

### Minigene reporter assay

The wild-type and variant amplicons encompassing intron 7, exon 8, and intron 8 of *ANOS1* (NC_000023) were obtained from the genomic DNA of the father and proband, respectively, using the following primers:

- F: 5′—CCGCTCGAGGCAGTCAGGAGCCACCGC—3′
- R: 5′—CTAGCTAGCCTCTCCCTCCATTGTGCCTTG—3′

The PCR products were cloned into the pSPL3 vector, which was kindly provided by Professor Leping Shao, The Affiliated Qingdao Municipal Hospital of Qingdao University, China) by XhoI and NheI. Wild-type (pSPL3-ANOS1-WT) and variant (pSPL3-ANOS1-MUT) constructs were verified by Sanger sequencing (data not shown). Human embryo kidney cells (HEK293) were transfected with the plasmid of pSPL3-ANOS1-WT or pSPL3-ANOS1-MUT for 24 h using jetPEI®DNA transfection Reagent (Polyplus, France) according to the manufacturer’s protocol. cDNA was obtained from RNA extraction by reverse-transcription polymerase chain reaction (RT-PCR). The cDNA products were used as templates for PCR amplification using the following pSPL3 vector-specific primers [12]:

- F: 5′—TCTGAGTCACCTGGACAACC—3′
- R: 5′—ATCTCAGTGGTATTTGTGAGC—3′

Splicing transcripts were identified by Sanger sequencing.

### Pathogenicity analysis of the variation

All mutations were assessed according to the American College of Medical Genetics and Genomics (ACMG) guideline. The pathogenicity of missense mutation was predicted using six tools: Revel (https://sites.google.com/site/revelgenomics), SIFT (http://sift.jcvi.org), PROVEAN (http://provean.jcvi.org), PolyPhen-2 (http://genetics.bwh.harvard.edu/pph2), MutationTaster (https://www.mutationtaster.org), M-CAP (http://bejerano.stanford.edu/mcap/), and GERP (http://mendel.stanford.edu/sidowlab/downloads/gerp/index.html). The evaluation of anosmin-1 amino acid conservation was performed by Ugene software [13] using the data from HomoloGene (ID:55,445). CNVs were evaluated according to the technical standards recommended by the ACMG and the Clinical Genome Resource (ClinGen).

## Results

### Monogene mutation screening and pedigree analysis

**Fig. 1:**
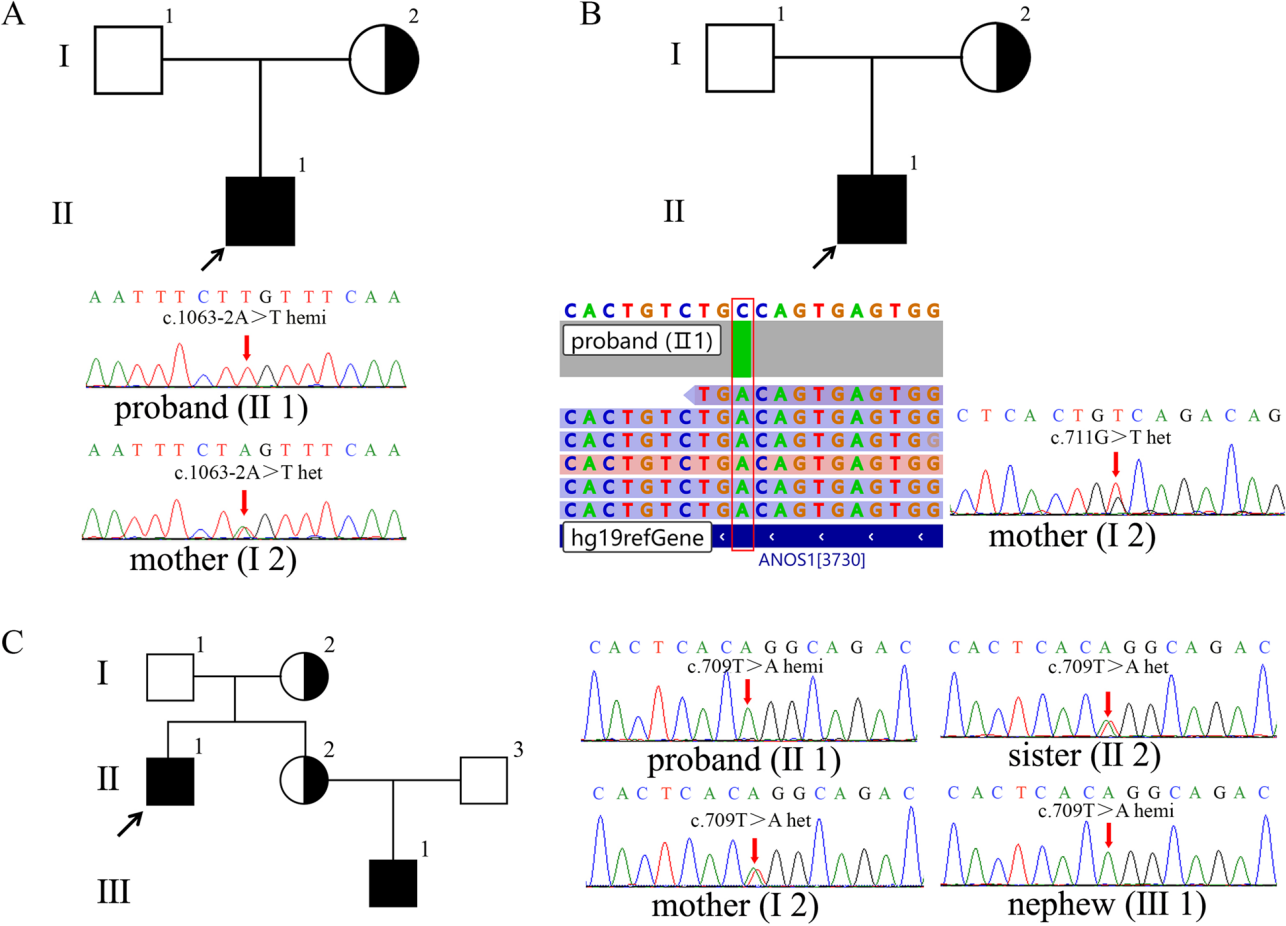
Pedigree and sequencing results of families 1–3. The pedigree showed core family members: square symbol represented males; circle symbol indicated females; Roman numerals represented generations; and Arabic numerals indicated the position of each individual within the family. **A** Pedigree and sequencing results of family 1. Sanger sequencing showed that the proband (II1) carried a hemizygous *ANOS1* c.1063-2 A > T mutation, while the proband’s mother (I2) carried a heterozygous *ANOS1* c.1063-2 A > T mutation. **B** Pedigree and sequencing results of family 2. NGS showed that the proband (II1) carried a hemizygous *ANOS1* c.711 G > T mutation, while Sanger sequencing showed that the proband’s mother (I2) carried a heterozygous *ANOS1* c.711 G > T mutation. **C** Pedigree and sequencing results of family 3. Sanger sequencing showed that the proband (II1) carried a hemizygous *ANOS1* c.709 T > A mutation, the proband’s mother (I2) and sister (II2) carried a same heterozygous *ANOS1* c.709 T > A mutation, and the proband’s nephew (III1) carried a hemizygote *ANOS1* c.709 T > A mutation

Five cases out of 35 unrelated patients were detected positive for mutations by custom-panel sequencing or WES, and their family members were analyzed. In family 1, a hemizygote *ANOS1* (the transcript is NM_000216, hg19) mutation c.1063-2 A > T was found in the proband (Fig. 1A, II1), and the results of pedigree verification confirmed that the mutation was inherited from the proband’s mother (I2).

In family 2, a hemizygote *ANOS1* c.711 G > T mutation was found in the proband (Fig. 1B, II1), and the results of the pedigree verification confirmed that the mutation was inherited from the proband’s mother (I2).

In family 3, a hemizygote *ANOS1* c.709 T > A mutation was found in the proband (Fig. 1C, II1). The results of the pedigree verification showed that the proband’s mother (I2) and sister (II2) carried heterozygous *ANOS1* c.709 T > A mutation, and the proband’s nephew (III1) carried hemizygote *ANOS1* c.709 T > A mutation.

**Fig. 2:**
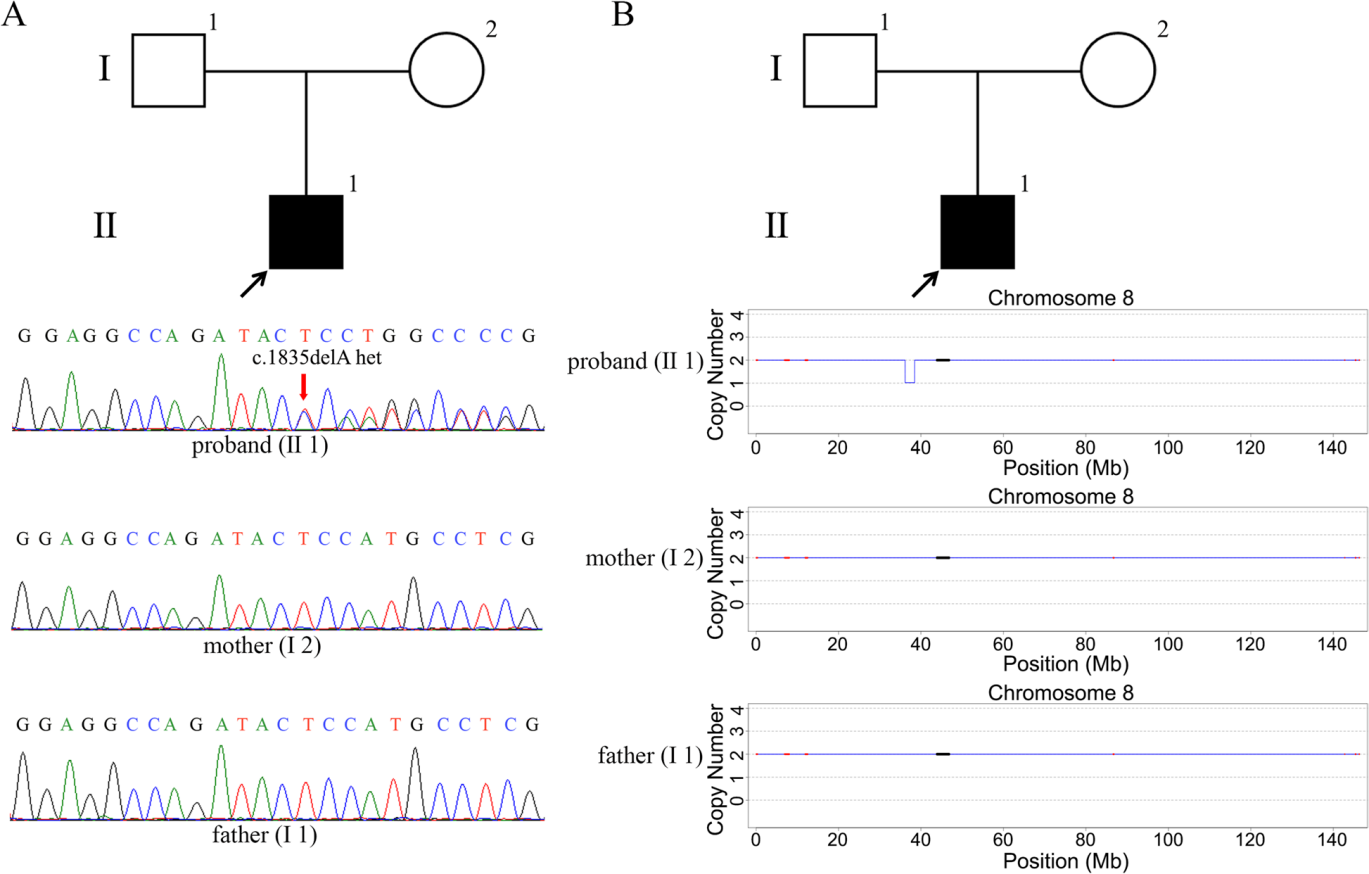
Pedigree and sequencing results of families 4 and 5. **A** Pedigree and sequencing results of family 4. Sanger sequencing showed that the proband (II1) carried a heterozygous *FGFR1* c.1835delA mutation, while the proband’s father (I1) and mother (I2) were normal. **B** Pedigree and CNV-seq results of family 5. CNV-seq results showed that the proband (II1) carried a 2.32 Mb deletion at 8p12-p11.22 (36,140,000–38,460,000), while the proband’s father (I1) and mother (I2) were normal

In family 4, a de novo heterozygous *FGFR1* (the transcript is NM_023110, hg19) mutation c.1835delA was found in the proband (Fig. 2A, II1), and the results of the pedigree verification showed that the father and mother were normal (I1 and I2).

In family 5, the custom-panel sequencing result showed a sporadic 1.68 Mb deletion at 8p11.23-p11.22, and the CNV-seq results showed a 2.32 Mb deletion at 8p12-p11.22 (36,140,000–38,460,000) in the proband (Fig. 2B, II1), while the father and mother were normal (I1 and I2).

### Verification of *ANOS1* c.1063-2 A > T splicing mutation ex vivo and in vitro

**Fig. 3:**
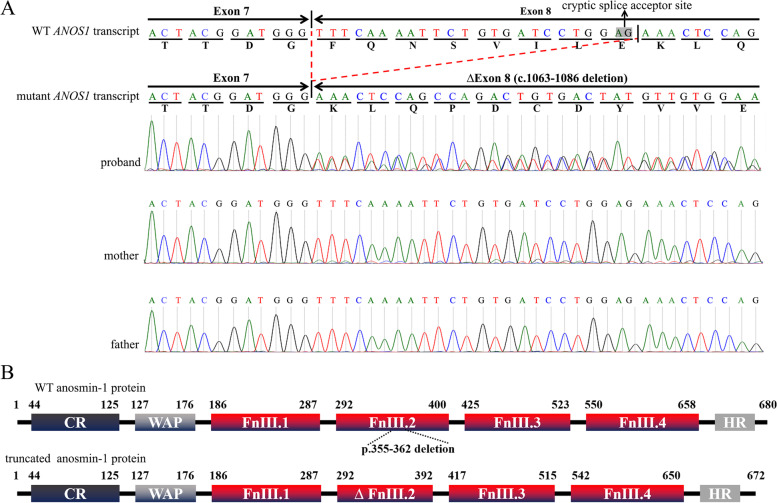
Results of the *ANOS1* mRNA transcripts analysis. **A** Sanger sequencing results of the RT-PCR products showed existence of both the wild-type (WT) transcript and the mutant transcript in the peripheral blood. The mutant *ANOS1* transcript was aberrant splicing with c.1063–1086 deletion. **B** Schematic structures of anosmin-1 proteins. The domains of anosmin-1 protein were retrieved from the Ensembl database (Transcript ID: ENST00000262648.8). The truncated protein exhibited a mutated FnIII.2 (ΔFnIII.2) domain with amino acid deletion at 355–362

To clarify the splicing effect of *ANOS1* c.1063-2 A > T mutation in family 1, cDNA sequence analysis was performed ex vivo. As shown in Fig. 3, c.1063-2 A > T mutation resulted in an aberrant splicing of 24-bp at the 5′ end of exon 8 and generated a new transcript with c.1063–1086 deletion; both of the normal and aberrant transcripts of *ANOS1* were detected in the proband’s peripheral blood (Fig. 3A). In addition, few aberrant transcripts were found in the maternal peripheral blood (Fig. 3A).

**Fig. 4:**
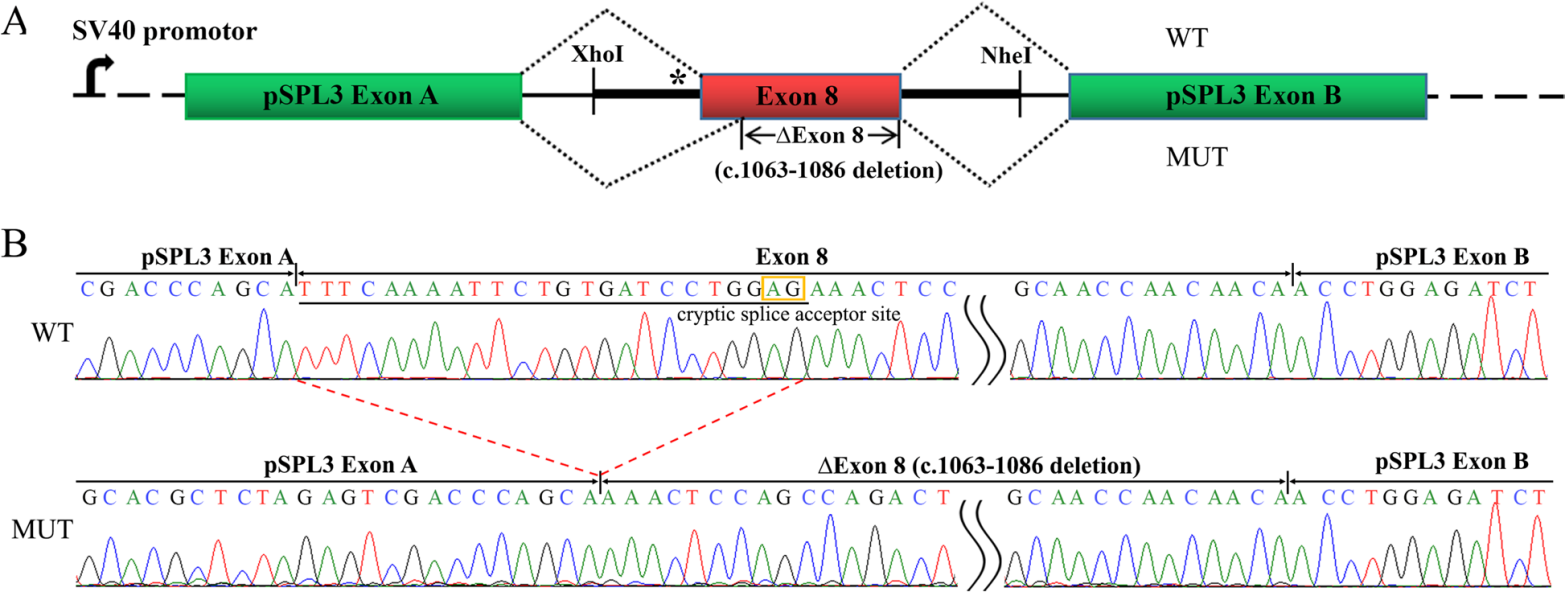
Results of minigene splicing assay. **A** Schematic diagram of minigene construction. The asterisk indicates the location of the c.1063-2 A > T mutation. The splicing pattern of wild-type (WT, top) and mutant (MUT, bottom) was showed respectively. **B** Minigene RT-PCR product sequencing results. The WT minigene formed normal mRNA composed of exon A, exon 8 and exon B. The mutant minigene caused a splicing abnormality, resulting in the 24 bp nucleotides deletion of 5′ end of exon 8 (c.1063–1086 deletion)

Splicing experiment in vitro was performed by constructing a splicing reporter minigene vector. As shown in Fig. 4B, the RT-PCR product sequence of the mutation construct was shorter than that of the wild-type construct, and the sequencing analysis revealed that the c.1063-2 A > T mutation led to an aberrant splicing consistent with the validation ex vivo. As expected, a truncated protein with p.355–362 deletion was generated (Fig. 3B).

### Pathogenicity analysis of *ANOS1* c.709 T > A and c.711 G > T missense mutations

*ANOS1* c.711 G > T and c.709 T > A variations in family 2 and 3 were two missense mutations that led to different amino acid substitutions of p.W237R and p.W237C, respectively, at the same position of anosmin-1 protein. The two novel variants had not been presented in the normal population by searching databases (1000 Genomes, EXAC, ESP6500, gnomAD, and dbSNP) (Table S2), and the W239 of anosmin-1 is highly conserved in multiple species (Fig. S1). Both p.W237R and p.W237C were predicted to be damaging using multiple software programs, such as Revel, SIFT, PolyPhen-2, MutationTaster, M-CAP, GERP, and PROVEAN (Table S2).

### Pathogenicity analysis of *FRFR1* mutations

**Fig. 5:**
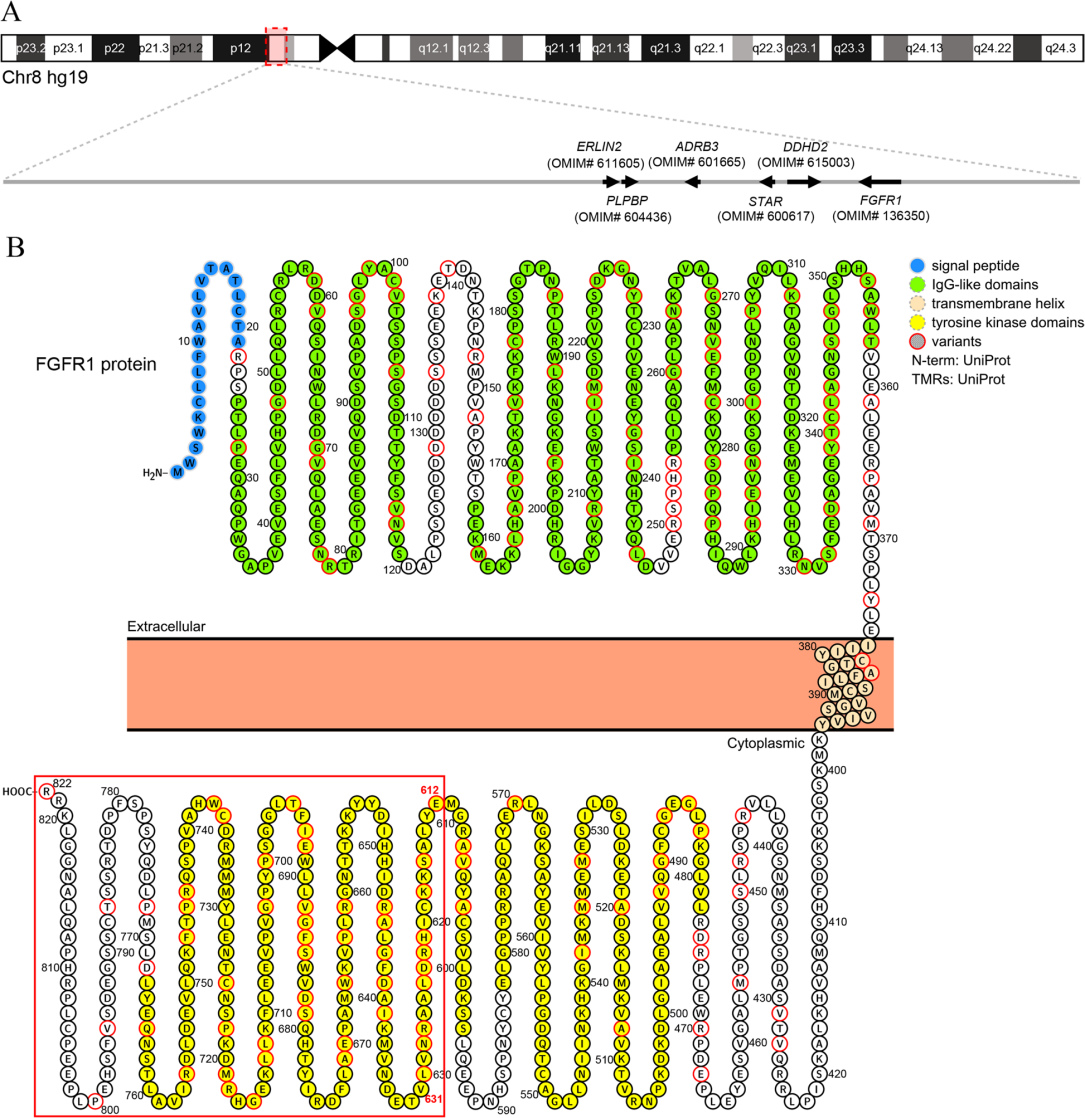
Chromosome 8p12-p11.22 gene map and the topological structure of FGFR1. **A** The orange box in the chromosome 8 diagram at the top indicated the region highlighted below. The OMIM morbid genes are listed at the bottom. **B** Membrane topology structure of full length FGFR1. Each circle represented a residue with the one-letter symbol. The different domains were labeled as indicated. Locations of pathogenic variations were colored as indicated. and the red box marked the amino acids that affected by *FRFR1* c.1835delA mutation

*FRFR1* c.1835delA variation in family 4 was a frameshift mutation that resulted in conversion of glutamate-612 to glycine and generated a stop codon at amino acid 631 (p.Glu612Glyfs*20). According to the ACMG guideline, *FRFR1* c.1835delA was assessed as a pathogenicity mutation using the evidence of PVS1 (null variant), PS2 (de novo variant), and PM2 (absent from controls). *FRFR1* p.Glu612Glyfs*20 mutation could express a truncated protein lacking the second tyrosine kinase domain (TKD) (Fig. 5B).

The CNV of seq[GRCh37]del(8)(p12p11.22)chr8:g.36140000_38460000del in patient 5 contained 19 protein-coding genes, including 6 morbid genes of the Online Mendelian Inheritance in Man (OMIM) database (Fig. 5A). The CNV was judged as a pathogenic variation using the evidence of 2A (complete overlap of an established haploinsufficiency gene *FGFR1* which contributes to KS2 [ClinGen ID: ISCA-32176], 1.00 point) according to the ACMG & ClinGen technical standards.

## Discussion

IHH is an inheritable disorder with clinical and genetic heterogeneity. The major pathogenesis of IHH is failure to activate pulsatile secretion of gonadotropin-releasing hormone (GnRH) during puberty [14]. As the main category of IHH, KS exhibits the typical characteristics of gonadal dysplasia and anosmia [15]. Complex symptoms hinder clinicians from making an accurate diagnosis of KS, while ultrasonography, magnetic resonance imaging, and serum hormone level measurements are routine auxiliary diagnostic methods. To date, more than 20 pathogenic genes have been found to be associated with KS, including *ANOS1*, *FGFR1*, *PROK2*, *PROKR2*, *NELF*, *KISSR1*, *CHD7*, *SEMA3A*, and *FGF8* [16]. Genetic analysis is the core diagnostic method of KS.

Anosmin-1 encoded by *ANOS1* plays important roles in substrate adhesion and cell migration of GnRH-1 neurons, axon outgrowth, and collateral formation, which is basically related to the central nervous system [17–19]. Herein, we reported three novel *ANOS1* mutations. One of them, *ANOS1* c.1063-2 A > T mutation in family 1 led to the production of a new transcript with c.1063–1086 deletion by the transcriptional splicing verification, and a truncated protein with p.355–362 deletion was predicted. It has been known that mutations that affect the splice donor and acceptor site (canonical GT-AG) are highly predictive of splicing defects, and always lead to an abnormal transcript with complete skipping of the downstream exon [20]. However, it is also possible that some splice acceptor site mutations disrupt the original splice acceptor site, and activate a cryptic splice acceptor site, resulting in an aberrant splicing with partial deletion of the downstream exon [21]. Therefore, the verification of a novel splice site variant is essential to clarify the pathogenic mechanism. In the present study, *ANOS1* c.1063-2 A > T mutation broken the splice acceptor site AG at intron 7, substituted by TG, and activated a cryptic splice acceptor site AG in exon 8. Intriguingly, both normal and aberrant transcripts of *ANOS1* were detected in the proband’s peripheral blood. Previous studies have reported that males carrying a hemizygous splicing mutation of genes located on chromosome X showed both normal and aberrant transcripts, such as c.5786 + 4 A > G in *ATRX*, c.1044 + 5 G > A in *FOXP3*, and c.463-6 T > G in *OGT* [22–24], so far the underlying mechanism remained unclear. We speculated that the introduced non-canonical splice site GT-TG caused by the c.1063-2 A > T mutation could produce the normal transcript as GT-TG splice site had been found in animal genomes [25]. Moreover, skewed X-chromosome inactivation (XCI) may be the reason of the few aberrant transcripts in the maternal peripheral blood. Polla et al*.* reported that the aberrant transcript of *MED12* in the peripheral blood samples of those heterozygous women could not be detected because of the skewed XCI [26].

Additionally, we reported two novel missense mutations of p.W237C in family 2 and p.W237R in family 3, which changed the amino acid sequence at the same position of anosmin-1 protein to different amino acid substitutions. W237 was located within the FnIII.1 domain with a highly conservatism. Anosmin-1 is a component of the FGFR1 pathway [27]. WAP, FnIII.1 FnIII.3, and FnIII.4 domains of anosmin-1 were confirmed to interact with FGFR1 [28, 29], and relevant mutations such as N267K in FnIII.1, E514K and F517L in FnIII.3 were proved to influence their interaction [30]. p.W237C and p.W237R may also regulate the signal transduction of the FGFR1 pathway and lead to disease occurrence. Notably, the mutations carried by patients in families 2 and 3 led to different clinical manifestations. Both patients in family 3 showed unilateral deletion of the left kidney, while patients in family 2 had healthy kidney, which attracted our attention to study the relationship between anosmin-1 and kidney development. Approximately 10% of males with *ANOS1* pathogenic variants also showed unilateral renal agenesis [31]. Georgopoulos et al*.* proposed that the FnIII repeats of *ANOS1* might be essential for normal renal development [32], but the specific mechanism has not been fully elucidated. Our findings may provide a new idea for the mechanism of anosmin-1 involvement in kidney development.

*FGFR1* is responsible for an autosomal dominant form of KS. A full-length FGFR1 protein consists of an extracellular region, composed of three immunoglobulin-like domains (IgI, IgII, and IgIII) responsible for the receptor’s affinity and specificity to its ligands, a single hydrophobic membrane-spanning segment, and two cytoplasmic TKDs with tyrosine kinase activity (Fig. 5B) [33]. The analysis of the total 198 *FGFR1* missense mutations listed in the HGMD database showed no obvious characteristics. The overall mutation locations are relatively scattered, mainly in the IgG-like domains and intracellular TKDs, but rarely in the transmembrane region (Fig. 5B). Herein, we identified a novel frameshift *FGFR1* mutation c.1835delA (p.Glu612Glyfs*20) in family 4. Following stop codons mutations of *FGFR1* including p.R661* and p.Q680* were considered as pathogenic mutations, with protein truncation and nonsense-mediated mRNA decay (NMD) being the underlying mechanisms [34, 35]. Similarly, *FGFR1* p.Glu612Glyfs*20 mutation was supposed to result in the synthesis of a truncated inactive receptor lacking an essential portion of the catalytic TKD. Alternatively, the *FGFR1* mRNA containing the premature stop codon could be degraded through NMD, leading to haploinsufficiency. In addition, we reported a family (family 5) with a CNV covering the entire *FGFR1*. To our knowledge, the whole *FGFR1* gene deletion was previously reported in only seven cases of KS [9, 33, 36, 37]. By searching the ClinVar database, only six cases with complete *FGFR1* were included (VCV000147723, VCV000057114, VCV000150494, VCV000145616, VCV000060360, VCV000362858, VCV000282690, and VCV000395746). The ClinGen database revealed that the haploinsufficiency of *FGFR1* contributed to KS2. The 2.32 Mb deletion at 8p12-p11.22 (36,140,000–38,460,000) in the proband of family 5 covered the whole *FGFR1* gene. This rare CNV was judged as the pathogenic mutation of KS in this patient as a sporadic case, which is the first report of whole *FGFR1* deletion in Chinese population.

## Conclusions

In this study, we identified three novel pathogenic mutations of *ANOS1* and two novel mutations of *FGFR1* in five Chinese families. In silico prediction indicated that *ANOS1* c.711 G > T and c.709 T > A were pathogenic mutations. Splicing experiments elucidated the pathogenesis of the *ANOS1* c.1063-2 A > T mutation. *FRFR1* c.1835delA mutation and del(8)(p12p11.22)chr8:g.36140000_38460000del were assessed as pathogenic variations. Therefore, our study expands the spectrum of mutations associated with KS and provides diagnostic evidence for patients who carry the same mutation in the future.

## Supplementary Information

**Additional file 1:** **Fig. S1 **Conservation analysis of anosmin-1. Assessment of amino acid conservation of anosmin-1 using Ugene. W237 of anosmin-1 (indicated in a black box) is highly conserved across various species.

**Additional file 2: Table S1** The clinical and genetic data of patients.

**Additional file 3: Table S2** The results of population databases retrieval and pathogenicity prediction for *ANOS1* gene c.709 T > A and c.711 G > T variation.

## Data Availability

The data analyzed during the current study is available from the corresponding author on reasonable request.

## Abbreviations

KS: Kallmann syndrome
IHH: Idiopathic hypogonadotropic hypogonadism
NGS: Next-generation sequencing
HGMD: Human Gene Mutation Database
FGFR1: Fibroblast growth factor receptor 1
DSD: Disorder of sex development
GnRH: Gonadotropin-releasing hormone
XCI: X-chromosome inactivation
TKD: Tyrosine kinase domain
NMD: Nonsense-mediated mRNA decay
