# Supplementary figures and images for: Mutation spectrum of Kallmann syndrome: identification of five novel mutations across ANOS1 and FGFR1
Source: Reprod Biol Endocrinol. 2023 Mar 1;21:23. doi: 10.1186/s12958-023-01074-w (PMC9976430; doi:10.1186/s12958-023-01074-w)

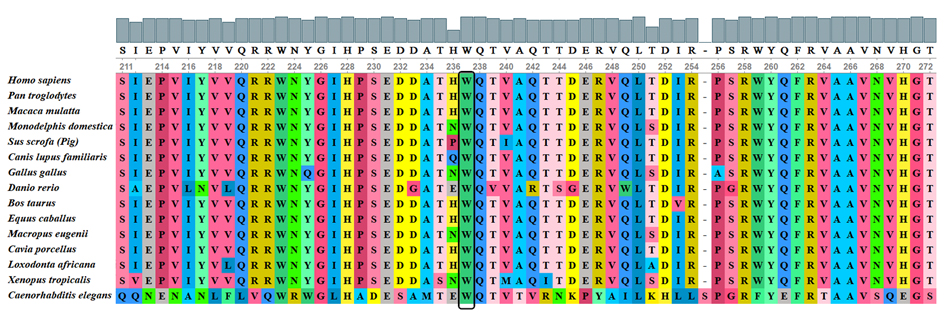

Supplement: Supplementary file 1 — Additional file 1: Fig. S1 Conservation analysis of anosmin-1. Assessment of amino acid conservation of anosmin-1 using Ugene. W237 of anosmin-1 (indicated in a black box) is highly conserved across various species. [file 12958_2023_1074_MOESM1_ESM.jpg]
