# Supplementary material for: Mutation spectrum of Kallmann syndrome: identification of five novel mutations across ANOS1 and FGFR1
Source: Reprod Biol Endocrinol. 2023 Mar 1;21:23. doi: 10.1186/s12958-023-01074-w (PMC9976430; doi:10.1186/s12958-023-01074-w)
Supplement: Supplementary file 2 — Additional file 2: Table S1 The clinical and genetic data of patients. [file 12958_2023_1074_MOESM2_ESM.docx]

**Table S1** The clinical and genetic data of patients.

| Patient | Karyotype | Phenotype | Test | Gene | variants |
| --- | --- | --- | --- | --- | --- |
| 1 | 46,XY | small penis, cryptorchidism, and testicular dysplasia | Custom-panel sequencing | *ANOS1* | c.1063-2A>T (splicing) |
| 2 | 46,XY | small penis, testicular dysplasia, obesity and gynecomastia | WES | *ANOS1* | c.711G>T (p.W237C) |
| 3 | 46,XY | cryptorchidism, left renal agenesis, and olfactory disorder | WES | *ANOS1* | c.709T>A (p.W237R) |
| 4 | 46,XY | small penis, olfactory disorder, testicular dysplasia and polydactylism | WES | *FGFR1* | c.1835delA (p.Glu612Glyfs*20) |
| 5 | 46,XY | cryptorchidism and testicular dysplasia | Custom-panel sequencing | *FGFR1* | deletion |
| 6 | 46,XY | hypospadias | Custom-panel sequencing | - | - |
| 7 | 46,XY | small penis | Custom-panel sequencing | - | - |
| 8 | 46,XY | small penis | Custom-panel sequencing | - | - |
| 9 | 46,XY | hypospadias | Custom-panel sequencing | - | - |
| 10 | 46,XY | hypospadias | Custom-panel sequencing | - | - |
| 11 | 46,XY | small penis | Custom-panel sequencing | - | - |
| 12 | 46,XY | cryptorchidism | Custom-panel sequencing | - | - |
| 13 | 46,XY | small penis and cryptorchidism | Custom-panel sequencing | - | - |
| 14 | 46,XY | small penis and cryptorchidism | Custom-panel sequencing | - | - |
| 15 | 46,XY | hypospadias | Custom-panel sequencing | - | - |
| 16 | 46,XY | hypospadias | Custom-panel sequencing | - | - |
| 17 | 46,XY | hypospadias and cryptorchidism | Custom-panel sequencing | - | - |
| 18 | 46,XY | hypospadias | Custom-panel sequencing | - | - |
| 19 | 46,XY | hypospadias | Custom-panel sequencing | - | - |
| 20 | 46,XY | hypospadias | Custom-panel sequencing | - | - |
| 21 | 46,XY | testicular dysplasia | Custom-panel sequencing | - | - |
| 22 | 46,XY | hypospadias | Custom-panel sequencing | - | - |
| 23 | 46,XY | hypospadias | Custom-panel sequencing | - | - |
| 24 | 46,XY | hypospadias | Custom-panel sequencing | - | - |
| 25 | 46,XY | hypospadias | Custom-panel sequencing | - | - |
| 26 | 46,XY | hypospadias | Custom-panel sequencing | - | - |
| 27 | 46,XY | small penis and hypospadias | Custom-panel sequencing | - | - |
| 28 | 46,XY | hypospadias | Custom-panel sequencing | - | - |
| 29 | 46,XY | small penis | Custom-panel sequencing | - | - |
| 30 | 46,XY | small penis | Custom-panel sequencing | - | - |
| 31 | 46,XY | testicular dysplasia | Custom-panel sequencing | - | - |
| 32 | 46,XY | small penis, testicular dysplasia | Custom-panel sequencing | - | - |
| 33 | 46,XY | hypospadias | Custom-panel sequencing | - | - |
| 34 | 46,XY | small penis and testicular dysplasia | Custom-panel sequencing | - | - |
| 35 | 46,XY | small penis, testicular dysplasia, cryptorchidism and cleft lip/palate | Custom-panel sequencing | - | - |

**-：No relevant variation.**
