# Supplementary material for: Mutation spectrum of Kallmann syndrome: identification of five novel mutations across ANOS1 and FGFR1
Source: Reprod Biol Endocrinol. 2023 Mar 1;21:23. doi: 10.1186/s12958-023-01074-w (PMC9976430; doi:10.1186/s12958-023-01074-w)
Supplement: Supplementary file 3 — Additional file 3: Table S2 The results of population databases retrieval and pathogenicity prediction for ANOS1 gene c.709 T > A and c.711 G > T variation. [file 12958_2023_1074_MOESM3_ESM.docx]

**Table S2** The results of population databases retrieval and pathogenicity prediction for *ANOS1* gene c.709T>A and c.711G>T variation.

|  | **Results** | |
| --- | --- | --- |
|  | **W237R** | **W237C** |
| 1000 Genomes | - | - |
| EXAC | - | - |
| ESP6500 | - | - |
| gnomAD | - | - |
| dbSNP | - | - |
| Revel | D (0.613) | D (0.674) |
| SIFT | Damaging (0.000) | Damaging (0) |
| PolyPhen-2 | Probably damaging (1.000) | Probably damaging (1.000) |
| MutationTaster | Disease causing (1) | Disease causing (1) |
| M-CAP | P (0.368) | P (0.831) |
| GERP | Conserved (4.15) | Conserved (4.15) |
| PROVEAN | Deleterious (-11.38) | Deleterious (-10.23) |

**Revel: Variations with a score higher than 0.75 are considered "damaging".**

**SIFT: Variations with a score below 0.05 are considered "damaging".**

**PolyPhen-2: Variations with a score locates in the interval of 0.909-1 are considered "probably damaging".**

**MutationTaster: PhastCons values vary between 0 and 1 and reflect the probability that each nucleotide belongs to a conserved element, based on the multiple alignment of genome sequences of 46 different species (the closer the value is to 1, the more probable the nucleotide is conserved).**

**M-CAP: . Variations with a score higher than 0.025 are considered "damaging".**

**GERP: Positive scores indicate a substitution deficit, while negative scores show that the site is probably evolving neutrally.**

**PROVEAN: Variations with a score equal to or below -2.5 are considered "deleterious".**

**-：not present.**
